# AraBAD Based Toolkit for Gene Expression and Metabolic Robustness Improvement in *Synechococcus elongatus*

**DOI:** 10.1038/s41598-017-17035-4

**Published:** 2017-12-22

**Authors:** Yi-Qi Cao, Qian Li, Peng-Fei Xia, Liu-Jing Wei, Ning Guo, Jian-Wei Li, Shu-Guang Wang

**Affiliations:** 1 School of Environmental Science and Engineering, Shandong University, 27 Shanda Nanlu, Jinan, 250100 China; 2 State Key Laboratory of Bioreactor Engineering, East China University of Science and Technology, 130 Meilong Road, Shanghai, 200237 China

## Abstract

As a novel chemical production platform, controllable and inducible modules in *Synechococcus elongatus* plus the ability of working in diurnal conditions are necessary. To the endeavors, inducible promoters, such as P_Trc_, have been refined from *Escherichia coli*, but the inducer isopropyl-β-D-thiogalactoside may cause several side-effects. Meanwhile, to promote the efficiency, photomixotrophic cultivation has been applied in *S*. *elongatus* with the additional organic carbon sources. In this study, we developed *L*-arabinose based modules consisted of both the P_BAD_ inducible promoter and the metabolism of *L*-arabinose in *S*. *elongatus*, since *L*-arabinose is an ideal heterologous feedstock for its availability and economic and environmental benefits. As expected, we achieved homogeneous and linear expression of the exogenous reporter through the P_BAD_ promoter, and the biomass increased in diurnal light condition via introducing *L*-arabinose metabolism pathway. Moreover, the combined AraBAD based toolkit containing both the P_BAD_ inducible module and the *L*-arabinose metabolism module could obtain gene expression and metabolic robustness improvement in *S*. *elongatus*. With the only additive *L*-arabinose, the novel strategy may generate a win-win scenario for both regulation and metabolism for autotrophic bio-production platforms.

## Introduction

Cyanobacteria have gained considerable popularity as a chemical production platform, which directly capture carbon dioxide and solar energy for bioconversions^1–5^. *Synechococcus elongatus* PCC7942 (thereafter *S*. *elongatus*) is a model cyanobacterium strain and prone to genetic modification for desired purposes. However, genetic control systems especially reliable inducible modules, which can boost production through precise regulation of heterologous gene expression, are limited in *S*. *elongatus* ^6–8^. Meanwhile, *S*. *elongatus* is a type of obligate photoautotrophs, and biomass accumulation is strongly dependent on the availability of light. Natural conditions containing the light and dark period should be explored to maximize productivity and lead to efficient utilization of resources to remain competitive^9–11^. These two aspects make both the synthetic biology tools development and products’ yields in *S*. *elongatus* lag behind what have been created in heterotrophic microbes.

Inducible genetic modules in *S*. *elongatus* have been investigated. However, characterized endogenous light sensitive promoter P_psbA2_, which controls transcription of photosystem genes, is undesirably sensitive to light and not active in darkness^12,13^. Endogenous transition metal responsive promoters P_idiA_ and P_Smt_, are difficult to use in practice for the complex off target regulatory system^14,15^. To realize artificial control of heterologous genetic networks in this typical autotrophic host, orthogonal genetic tool P_lacI_, has been successfully transplanted from *Escherichia coli* to substitute endogenous promoters in *S*. *elongatus* ^16^. In the system, the LacI repressor blocked the P_Trc_ promoter. The addition of inducer, isopropyl-β-D-thiogalactoside (thereafter IPTG), resulted in inactivation of LacI, derepression of the promoter and subsequent high-level expression of the insert gene^16^. However, IPTG is relative expensive^17^ and cannot be metabolized by cells. The residual IPTG will bring environmental issues. Besides, this promoter displays undesired baseline expression in *S*. *elongatus* ^4,18^, thus failing sophisticated regulations and making it no suited for the production of proteins that are toxic to the host.

The obligate photoautotrophic organism *S*. *elongatus*, has been engineered to produce various chemicals at higher titers and productivities relative to other cyanobacteria^4,19,20^. However, almost all chemical production studies in *S*. *elongatus* has exclusively conducted in continuous light, as cyanobacteria can only fix CO_2_ and accumulate biomass with light^21^. Natural sunlight is freely available, so it would be more feasible and commercial to utilize *S*. *elongatus* in natural conditions, where light and dark cycle every 12 h. To overcome this innate physiology, several studies explored the potentials of transplanting heterotrophic carbon metabolic pathways to enable sugar utilization. For instance, McEwen *et al*.^9^ successfully engineered *S*. *elongatus* to metabolize glucose and xylose via installing sugar transporters and peripheral metabolic processes. The engineered strains could produce 2,3-butanediol at higher levels in diurnal condition^21^. These efforts shed light on converting the obligate photoautotrophic cyanobacterium *S*. *elongatus* to photomixotrophic platform to override the limitation of darkness.

As such, we would like to design a commercial and inducible promoter without leakage and enable *S*. *elongatus* to work in diurnal light condition with the 12 h day-night cycles using sugar inducers. As reported, the araBAD-based P_BAD_ promoter carrying the *araC-*P_BAD_ expression cassette has been successfully utilized for reliable gene expression in gram-negative bacteria, such as *E*. *coli* ^22^. This system is regulated by the transcriptional regulator AraC. In the absence of *L*-arabinose, the AraC dimer creates DNA loop and represses transcription from P_BAD_. The binding of *L*-arabinose to AraC changes the position of AraC dimer and releases the DNA loop, allowing transcription from P_BAD_^23^. The P_BAD_ promoter provides quite a low-level of expression leakage and promising dosage-dependent tight control of heterologous genetic modules^24^. Fortunately, the inducer for the P_BAD_ promoter, *L*-arabinose, is also one of the most common pentose in hemicelluloses^25^, which may also have the potential to be the feedstock for microbial production of bio-chemicals in *S*. *elongatus* under diurnal condition. Recently, the P_BAD_ module was established in another type of model cyanobacteria, *Synechocystis* sp. PCC 6803, expanding the tools available for transcriptional regulation^26^. However, this sensor has not been applied in *S*. *elongatus*, and the effects of *L*-arabinose and *L*-arabinose utilization pathways on *S*. *elongatus* remains unknown.

To expand synthetic biology toolboxes and to enable *S*. *elongatus* to accumulate biomass in diurnal condition, the current study proposed an applicable AraBAD based toolkit in *S*. *elongatus* including both the inducible module driven by P_BAD_ promoter and *L*-arabinose metabolic pathway. In this case, *L*-arabinose was not only the inducer but also a carbon source to regulate genetic modules and promote the metabolic robustness. Moreover, this designed toolkit was economic and environmental sound. We believe that our strategy provides a preferable genetic toolkit for *S*. *elongatus* and generates a win-win scenario for both gene expression and metabolic robustness in autotrophic bio-production platforms.

## Results and Discussion

### P_Trc_ and P_BAD_ led gene expression in *S*. *elongatus*

An ideal promoter system should exhibit no leaky expression and allow linear control of the gene expression levels via exerting different concentrations of inducers^27^. So, we first compared the P_Trc_ and P_BAD_ promoters in *S*. *elongatus* using a modified GFP (mtGFP)^28^ as a reporter because of a stronger fluorescence than eGFP (Fig. S1). Both P_Trc_-*mtgfp* and P_BAD_-*mtgfp* fragments were integrated into the chromosome at neutral site I (NSI)^29^ in *S*. *elongatus*. Relative fluorescence units (RFU) of the cultures were standardized as fluorescent intensity (RFU/OD_730_), indicating the mtGFP expression level per turbidity.

**Figure 1:**
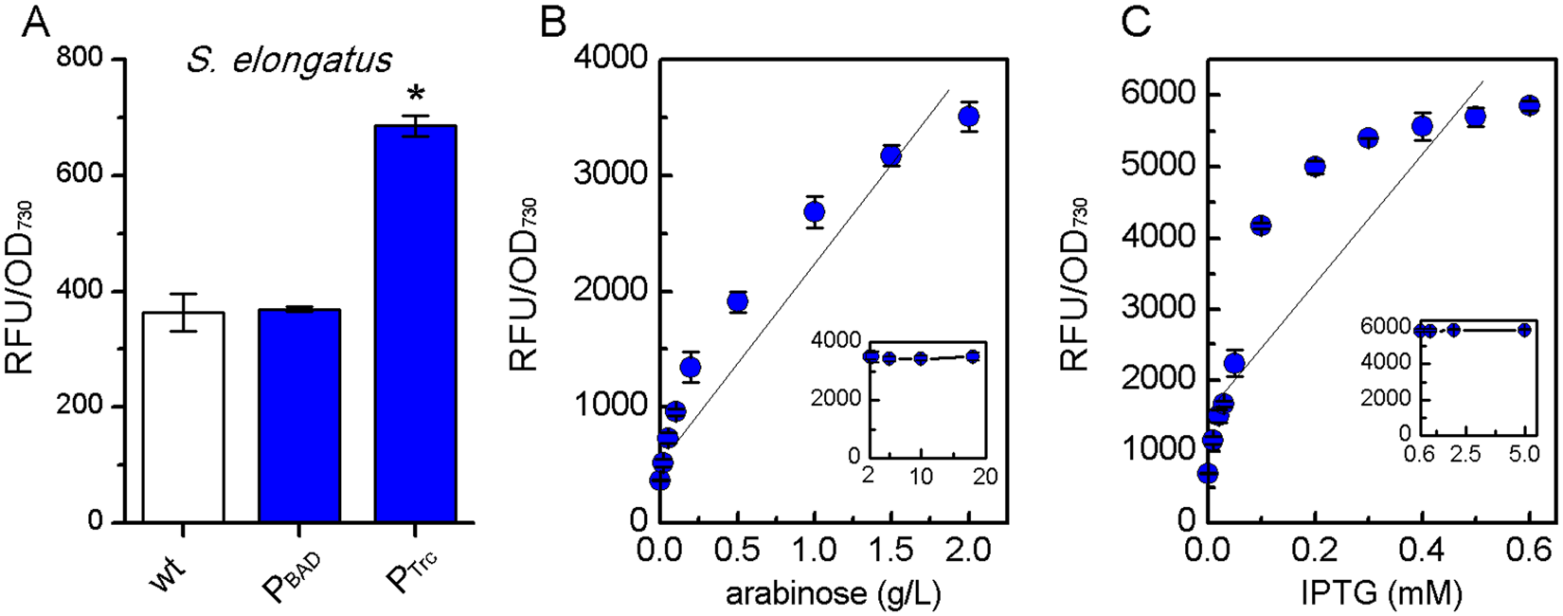
Fluorescence of mtGFP led by P_BAD_ and P_Trc_ promoters in *S*. *elongatus*. (**A**) Standardized fluorescent intensity (RFU/OD_730_) of wide-type strain, YQs1 (P_BAD_) and YQs2 (P_Trc_) under non-induced condition. An asterisk (*) represents a statistical difference (p < 0.05). (**B**) Linear relationship between the standardized fluorescent intensity (RFU/OD_730_) and *L*-arabinose of the P_BAD_ promoter in *S*. *elongatus*. (**C**) Linear relationship between the standardized fluorescent intensity (RFU/OD_730_) and IPTG of the P_Trc_ promoter in *S*. *elongatus*.

As shown in Fig. 1A, the leaky expression of the P_Trc_ is remarkable (ΔRFU/OD_730_ = 323), while the P_BAD_ promoter showed almost no leaky expression (ΔRFU/OD_730_ = 7), though P_Trc_ promoter exhibited stronger expression of mtGFP than P_BAD_ in *S*. *elongatus*. This was in agreement with the result shown in *E*. *coli* strains that harboring the P_Trc_ and P_BAD_ promoters based on *S*. *elongatus* specialized plasmids pAM2991 backbone (Fig. S2). Besides, the arabinose sensors used in *Synechocystis* sp. PCC 6803, were also tightly off when not induced^26^. Moreover, the expression levels of mtGFP driven by P_BAD_ varied linearly (R^2^ = 0.935, Fig. 1B) along with the extracellular concentrations of *L*-arabinose (0–2 g/L) after inducing for 2 days. The mtGFP expression led by P_BAD_ stopped to increase with extra *L*-arabinose, suggesting that the saturation concentration of *L*-arabinose was 2 g/L for P_BAD_ in *S*. *elongatus* (Fig. 1B). However, correlation between IPTG concentrations and expression levels of mtGFP led by P_Trc_ promoter, did not show a promising linear manner within the unsaturated concentration scope of 0–0.6 mM IPTG (R^2^ = 0.766, Fig. 1C). This result indicated the difficulty of sophisticated gene regulation via P_Trc_ promoter. Thus, the P_BAD_ promoter provides a more reliable regulation tool for gene expression than the P_Trc_ promoter in *S*. *elongatus*.

**Figure 2:**
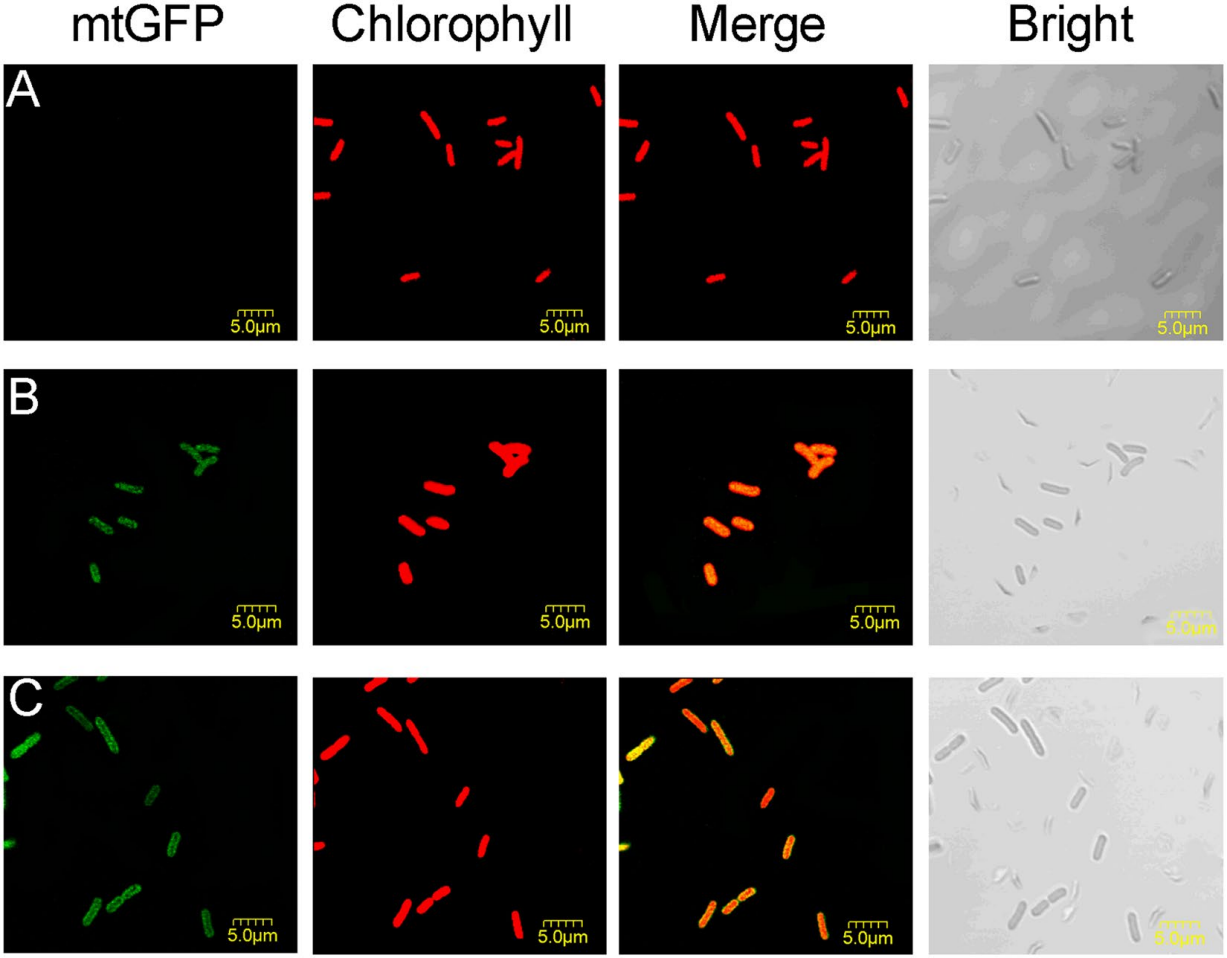
Confocal laser scanning microscope images of mtGFP expression in *S*. *elongatus*. (**A**) The wide-type *S*. *elongatus*. (**B**) Fluorescence of mtGFP driven by P_BAD_ promoter with 2 g/L *L*-arabinose after inducing for 2 days. (**C**) Fluorescence of mtGFP driven by P_Trc_ promoter with 1 mM IPTG after inducing for 2 days.

Apart from that, the expression of mtGFP was also recorded in individual cells by the confocal laser scanning microscope (thereafter CLSM). For wild-type *S*. *elongatus*, it showed only red fluorescence but no green fluorescence with the correspondent filters (Fig. 2A). The red fluorescence represented the native chlorophyll that emitting in the wavelength coverage. When mtGFP driven by the P_BAD_ promoter was induced in *S*. *elongatus*, the result showed homogeneous green fluorescence, indicating the homogeneous expression of mtGFP across cells (Fig. 2B). The transport of *L*-arabinose might attribute to unknown native transporters in *S*. *elongatus*. For the P_Trc_ promoter in *S*. *elongatus*, mtGFP fluorescence of the cells was not homogeneous but randomly across cells (Fig. 2C). It may result from the diverse amount of inducers inside the cells that bind to the repressor LacI^30^. Moreover, as imaged by the fluorescence microscope, IPTG at higher concentration (5 mM) led to morphological changes, while high concentration of the inducer *L*-arabinose showed no harm to the shape of *S*. *elongatus* even when it reached 20 g/L (Fig. S3).

### Heterogeneous *L*-arabinose metabolism pathway for biomass increase in *S*. *elongatus*

We achieved homogeneous and linear gene expression through P_BAD_ promoter in *S*. *elongatus*. Though *L*-arabinose did no harm to the shape of *S*. *elongatus*, the addition of *L*-arabinose could not improve but inhibited the growth by almost 30% after 10 days cultivation (Fig. S4). It putatively resulted from the lack of *L*-arabinose metabolism in *S*. *elongatus*, and the accumulated intracellular *L*-arabinose caused the metabolic stress. To test the metabolic burden of *L*-arabinose, we measured the chlorophyll content, a type of potential indicators of metabolic stress^31^. As assumed, intracellular chlorophyll content was decreased by 20% upon the addition of 2 g/L *L*-arabinose (Fig. 4D).

Then, we hypothesized that the inhibitory effects of *L*-arabinose could be alleviated by introducing the *L*-arabinose utilization pathway. As reported, xylose can be metabolized by *S*. *elongatus* with heterologous expression of *xylE* and *xylAB* that encoding for the *E*. *coli* xylose transporter and the first two steps of xylose utilization^9^. The result suggests that introducing the *L*-arabinose pathway in *S*. *elongatus* may enable the strain to metabolize *L*-arabinose. Moreover, introducing the sugar metabolic pathway brought another bonus, and it could enable the growth of *S*. *elongatus* in the darkness, thus increasing cellular robustness for bio-production^9–11,21^. As such, we planned to transplant the entire *L*-arabinose metabolic process from *E*. *coli* to *S*. *elongatus*.

**Figure 3:**
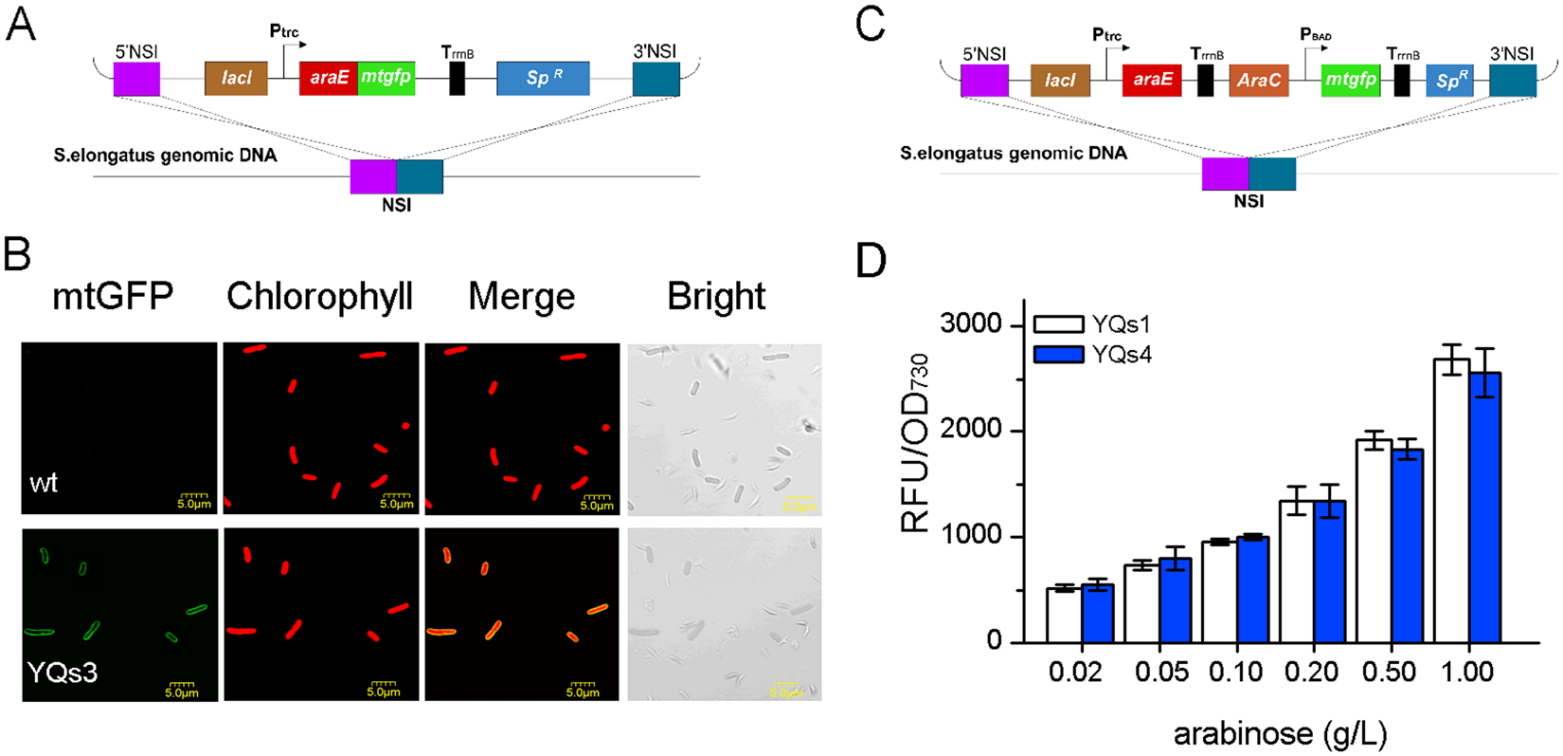
Exploration of the *L*-arabinose transport ability in *S*. *elongatus*. (**A**) The designing scheme of *araE-mtgfp* fragment integrated into *S*. *elongatus* genome (YQs3). (**B**) CLSM images of wide-type *S*. *elongatus* and YQs3. (**C**) The designing scheme of *araE* and *P*_BAD_ *-mtgfp* fragments integrated into *S*. *elongatus* genome (YQs4). (**D**) The expression level of mtGFP in YQs1 and YQs4 after inducing with unsaturated concentrations of *L*-arabinose for 2 days.

First, we tested the effects on *S*. *elongatus* of the *E*. *coli* AraE, which is one of the major facilitator superfamily (MFS) transporters^32^. To do so, the *mtgfp* was fused to the 3′ end of *araE* gene, generating *araE-mtgfp*, and it was then inserted into the *S*. *elongatus* chromosome under the control of P_Trc_ (Fig. 3A). After induction with 1 mM IPTG for 2 days, cells were recorded by CLSM. Strain containing *araE-mtgfp* fragment showed green fluorescence on the cellular membrane of all cells, while the wide-type strain exhibited no green fluorescence, implied the functional expression and location of AraE in YQs3 (Fig. 3B). Next, to investigate whether *E*.*coli* AraE could lead more *L*-arabinose transport, we constructed YQs4 containing both the *araC-*P_BAD_ expression cassette and AraE transporter driven by P_Trc_ (Fig. 3C). As the results shown in Fig. 3D, there were almost no mtGFP expression level changes within unsaturated concentration of *L*-arabinose, indicating no increase of intracellular *L*-arabinose accumulation. Thus, the transport of *L*-arabinose in *S*. *elongatus* could not be further improved by *E*. *coli* AraE, and *S*. *elongatus* was able to transport sufficient *L*-arabinose with its native transporters.

**Figure 4:**
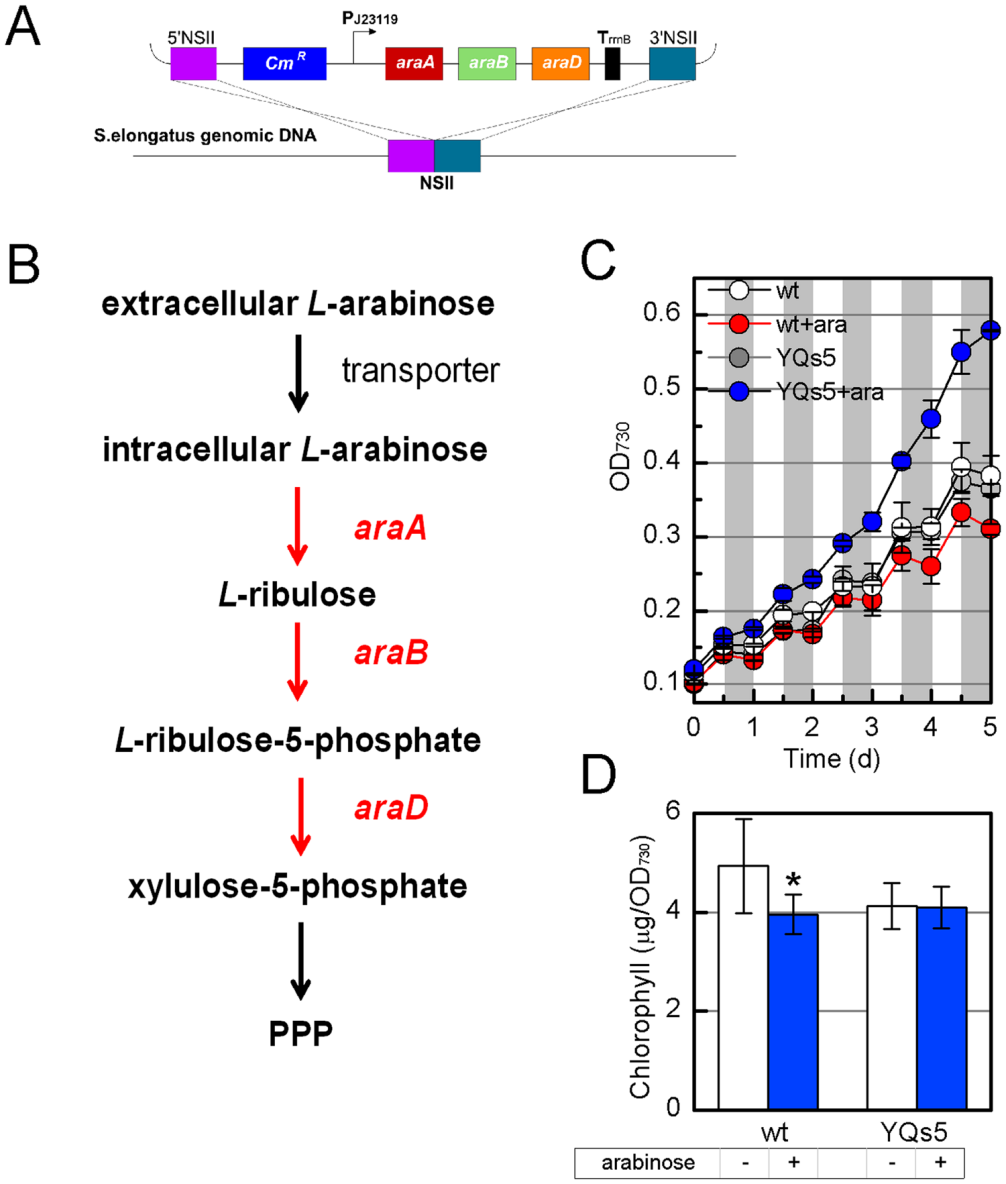
Installation of the *L*-arabinose degradation pathway. (**A**) The designing scheme of *L*-arabinose degradation pathway integrated into *S*. *elongatus* genome (YQs5). (**B**) Synthetic *L*-arabinose degradation pathway in *S*. *elongatus*. Red arrows indicate steps catalyzed by heterologous enzymes. PPP: pentose phosphate pathway. (**C**) Growth curve of the wide-type *S*. *elongatus* and YQs5 in diurnal condition with or without 2 g/L *L*-arabinose for 5 days. (**D**) Chlorophyll content of the wide-type *S*. *elongatus* and YQs5 with or without 2 g/L *L*-arabinose in constant light condition for 5 days cultivation. An asterisk (*) represents a statistical difference (p < 0.05).

Second, we installed the *E*. *coli L*-arabinose peripheral metabolic process into *S*. *elongatus* without AraE. In *E*. *coli*, intracellular *L*-arabinose is converted by arabinose isomerase (AraA) to *L*-ribulose, which is subsequently phosphorylated to *L*-ribulose-5-phosphate by *L*-ribulokinase (AraB) and then converted into xylulose-5-phosphate by *L*-ribulose-5-phosphate epimerase (AraD), where it enters the pentose phosphate pathway (PPP)^33^. So, the *araA*, *araB* and *araD* genes responsible for the first three steps of *L*-arabinose metabolic process from *E*. *coli* MG1655, were integrated into *S*. *elongatus* genome at neutral site II (NSII)^34^ to construct YQs5 (Fig. 4A). As shown in Fig. 4C, this operon allowed heterotrophic growth of YQs5 in the dark part of diurnal condition, indicating the *E*. *coli araBAD* genes were functional in *S*. *elongatus*. In addition, with the consumption of *L*-arabinose, there was no existence of *L*-arabinose metabolic stress in YQs5 (Fig. 4D). Thus, in order to override the metabolic stress and increase biomass in diurnal conditions, it is essential to introduce the heterogeneous *L*-arabinose metabolic pathway into *S*. *elongatus*.

### Time-course performance of AraBAD based toolkit in *S*. *elongatus*

**Figure 5:**
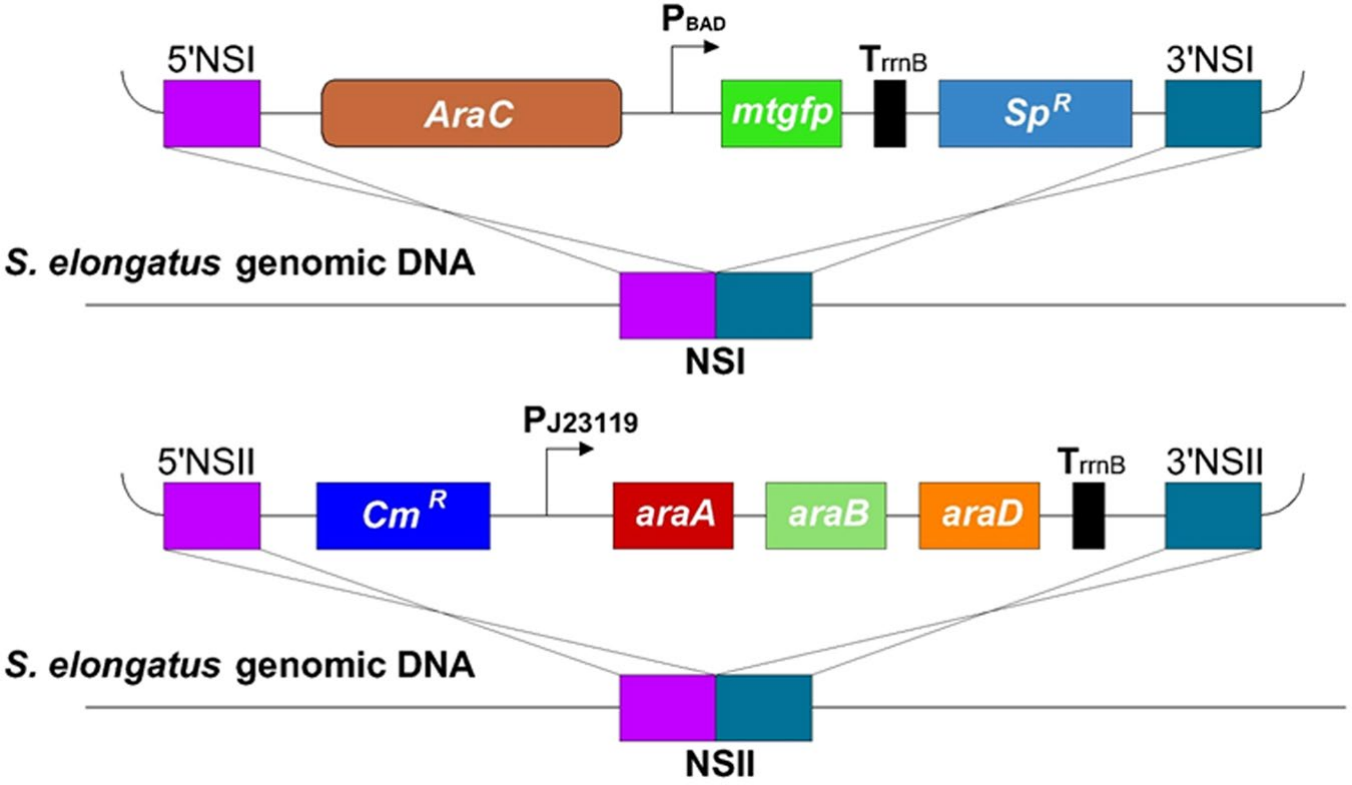
The designing scheme of AraBAD toolkit in *S*. *elongatus* genome covering both the P_BAD_-*mtgfp* fragment integrated into NSI and *L*-arabinose degradation pathway integrated into NSII (YQs6).

We have successfully applied P_BAD_ promoter in *S*. *elongatus* and engineered *S*. *elongatus* to metabolize *L*-arabinose. To achieve genetic control and promote metabolic robustness with the only inducer *L*-arabinose, we next built YQs6 that harboring the combined AraBAD toolkit in *S*. *elongates*. The toolkit contained both the P_BAD_ inducible and the *L*-arabinose metabolism modules (Fig. 5). Because the strength of the P_BAD_ promoter was strongly dependent on the *L*-arabinose concentration, and the metabolism of *L*-arabinose will affect the residual concentration. It is necessary to explore the relationship between the regulation and metabolism. Thus, the growth rate, *L*-arabinose metabolism and kinetic of the mtGFP expression were further analyzed. The saturation concentration of *L*-arabinose for the P_BAD_ promoter (2 g/L) in *S*. *elongatus* was chosen for the study.

**Figure 6:**
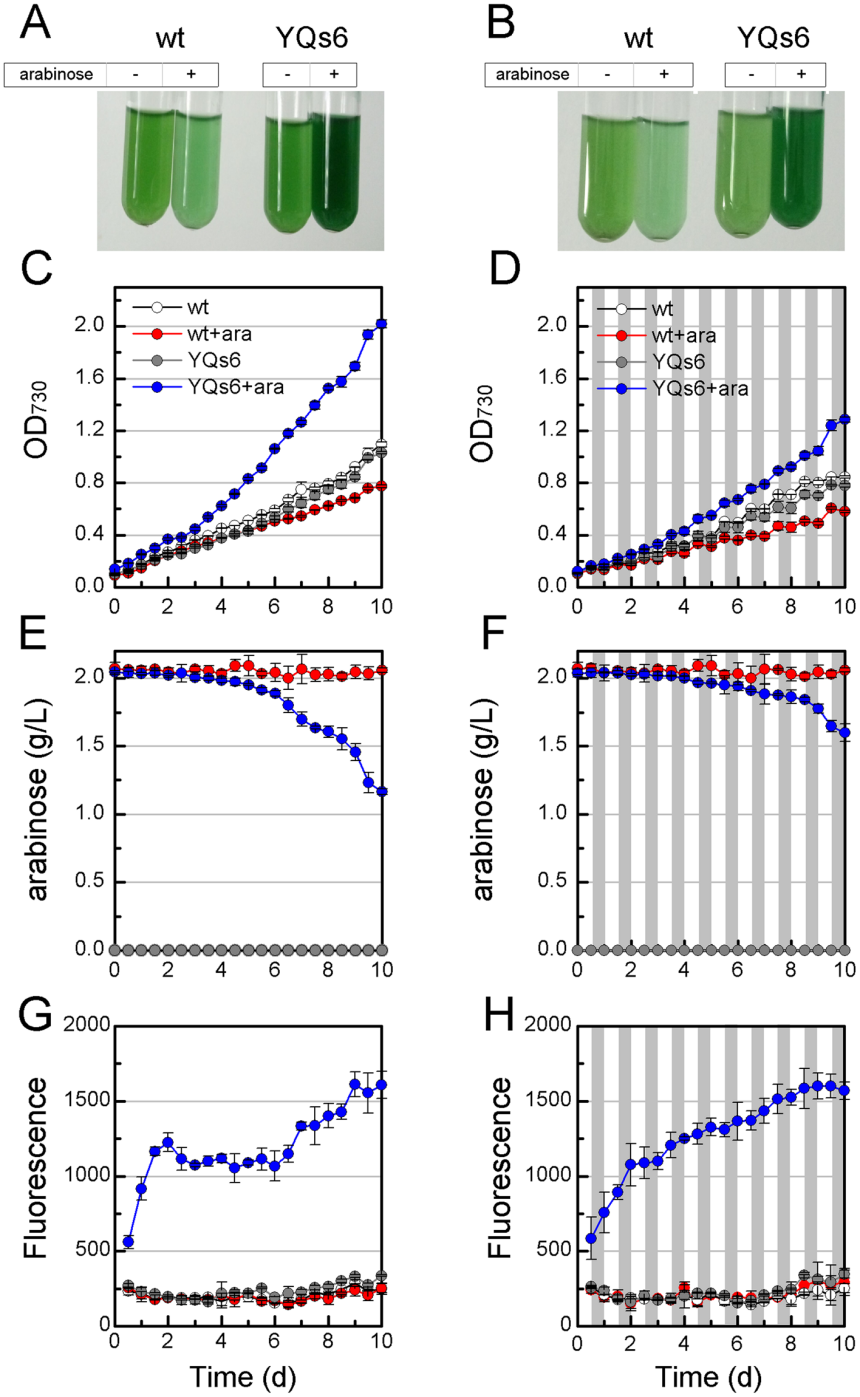
Time-course performance of the AraBAD based toolkit in *S*. *elongatus*. Growth situation of the wide-type *S*. *elongatus* and YQs6 with or without 2 g/L *L*-arabinose in (**A**) continuous and (**B**) diurnal conditions in the tenth day. Growth curve of the wide-type *S*. *elongatus* and YQs6 in (**C**) continuous and (**D**) diurnal conditions for 10 days. *L*-arabinose utilization of the wide-type *S*. *elongatus* and YQs6 in (**E**) continuous and (**F**) diurnal conditions for 10 days. Kinetic of the total mtGFP fluorescence of 200 μL culture broth in (**G**) continuous and (**H**) diurnal conditions for 10 days.

As shown in Fig. 6, YQs6 could get extremely high growth rate and reporter expression with the addition of *L*-arabinose, indicating both metabolism robustness improvement and the P_BAD_ inducible module operation can be achieved at the same time. The growth rate of YQs6 quickly increased and reached OD_730_ 1.286 in diurnal condition in the tenth day, and it could even reach 2.020 in continuous light condition. Nevertheless, the wide-type *S*. *elongatus* with *L*-arabinose could only grow to OD_730_ of 0.774 in continuous light condition (Fig. 6C and D). Thus, YQs6 exhibited an improved ability to sequester carbon than the wide-type *S*. *elongatus*. It could also be identified from Fig. 6E and F, the *L*-arabinose consumption was not detected in wide-type *S*. *elongatus*, but for YQs6, the consumption of *L*-arabinose was 0.402 g/L in diurnal condition and 0.836 g/L in continuous light condition after 10 days cultivation. Meanwhile, the total mtGFP fluorescence, representing the overall expression level per unit volume (200 μL), increased because of the improvement of cell amounts, though the concentration of *L*-arabinose got decreased (Fig. 6G and H). With the addition of *L*-arabinose, the fluorescence increased quickly over the first 2 days and the increase slowdown afterwards (Fig. 6G and H). The expression change of mtGFP may rely on the consumption of *L*-arabinose, as the strength of the P_BAD_ promoter is dependent on the intracellular *L*-arabinose concentration.

**Figure 7:**
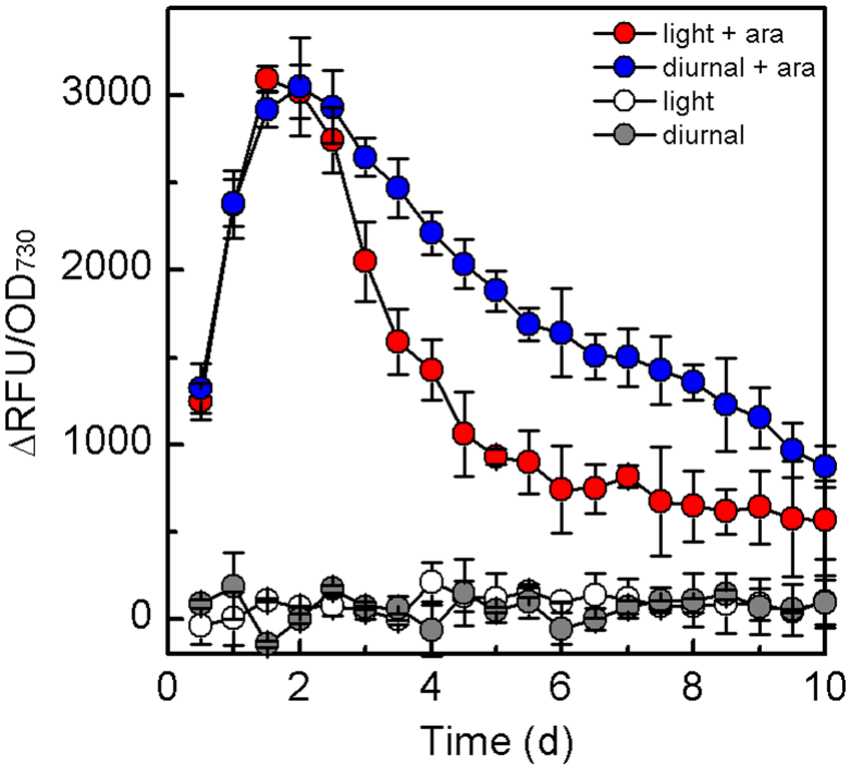
Relative standardized fluorescent intensity (ΔRFU/OD_730_) of YQs6 with or without 2 g/L *L*-arabinose added in BG-11 medium in continuous light and diurnal conditions. In diurnal condition, ΔRFU/OD_730_ = (RFU_YQs6_/OD_YQs6_)_diurnal_ − (RFU_wt_/OD_wt_)_diurnal_. In continuous light condition, ΔRFU/OD_730_ = (RFU_YQs6_/OD_YQs6_)_light_ − (RFU_wt_/OD_wt_)_light_.

To properly evaluate the AarBAD based toolkit in *S*. *elongatus*, the fluorescence intensity was normalized with the cell density (OD_730_), and ΔRFU/OD_730_ represented the difference of RFU/OD_730_ between YQs6 and wide-type strain (Fig. 7). Overtime, the P_BAD_ promoter was still tightly off in the absence of *L*-arabinose, no matter in light or dark condition (Fig. 7), and the result corresponded to the P_BAD_ promoter in *Synechocystis* ^26^. The normalized fluorescence of YQs6 quickly increased in the first 2 days, indicating that the inducing for the P_BAD_ toolkit to reach the maximum in *S*. *elongatus* needed about 2 days in both continuous light and diurnal conditions. Generally, in continuous light condition, YQs6 grew more quickly together with photosynthesis, and led the more degradation of *L*-arabinose and the lower fluorescence. It is necessary to emphasize that, though *L*-arabinose concentration change was not obvious from the second day to fifth day, the normalized fluorescence of YQs6 decreased lower in diurnal condition (Fig. 7). We considered that *L*-arabinose metabolism intracellular in the dark was weaker than in the light, leading a higher intracellular *L*-arabinose concentration for the P_BAD_ promoter. In summary, when using the AraBAD toolkit with natural sunlight, *S*. *elongatus* could achieve both a relatively high growth rate and genes expression level. Moreover, the residual inducer would be metabolized by *S*. *elongatus*, leading a less residual impact on the environment.

## Conclusion

In this article, we presented a novel strategy using the only additive *L*-arabinose for both gene expression and metabolic robustness improvement in *S*. *elongatus* with the AraBAD based toolkit. The P_BAD_ promoter in *S*. *elongatus* led precise control of reporter and homogenous expression across cells. Meanwhile, by introducing the *L*-arabinose metabolic pathway from *E*. *coli*, *S*. *elongatus* could both override the metabolic stress and achieve biomass increase for bio-production in the mixotrophic cultivation. The combined AraBAD based toolkit containing both the P_BAD_ inducible and *L*-arabinose metabolism modules, could realize a win-win scenario for both regulation and metabolism with the only one regulator. Future study might base on modifying a more appropriate AraBAD toolkit for *S*. *elongatus* by changing the ribosome binding site (RBS) or other genetic modules to improve the strength of the P_BAD_ promoter, and also, using the toolkit for bio-chemicals production at industrial level. In general, we believe this genetic toolkit will promote the utility and engineering of the autotrophic platform, *S*. *elongatus*, to use the natural sunlight for designed purposes with economic and environmental benefits.

## Methods

### Strains and culture conditions

*S*. *elongatus* PCC7942 was obtained from ATCC (American Type Culture Collection, ATCC #33912). All cyanobacterial strains were cultured in 250 mL conical flasks with a total volum of 100 mL BG-11 medum^35^ at 30 °C. The lumination was set to 2,000–3,000 Lux. Not other mentioned, 20 μg/mL spectinomycin and 7.5 μg/mL chloramphenicol were used for the mutant strains when necssery. All assays were conducted using biological triplicates. Cell gowth was monitored by measuring OD_730_ for each biological triplicate one time.

All *E*. *coli* strains were cultivated in the Luria-Bertain (LB) medium (10 g/L tryptone, 5 g/L yeast extract and 10 g/L NaCl) at 37 °C with shaking at 150 rpm. *E*. *coli* MG1655 was used as template to extract the genomic DNA, *E*. *coli* DH5α was used for cloning and manipulation of plasmids, and *E*. *coli* BW25113 was used as the testing platform. Ampicillin (100 μg/mL), spectinomycin (100 μg/mL) and chloromycetin (170 μg/mL) were added for the maintenance of the plasmids containing these antibiotic resistance markers. All strains used in this study were listed in Table 1.

**Table 1:** Strains used in this study.

Strains	Relevant Genotypes	Sources
*E*. *coli* strains		
BW25113	*E*. *coli* BW25113 (Δ*araBAD*_AH33_)	CGSC#7636
YQe1	Harboring pYQ5 (pAM2991, but *P*_BAD_-*mtgfp*)	this study
YQe2	Harboring pYQ2 (pAM2991, but *P*_Trc_: *mtgfp*)	this study
*S*. *elongatus* strains		
wide-type (wt)	*S*. *elongatus* PCC7942	S. S. Golden
YQs1	*P*_BAD_-*mtgfp* integrated at NSI	this study
YQs2	*P*_Trc_: *mtgfp* integrated at NSI	this study
YQs3	*P*_Trc_: *araE*-*mtgfp* integrated at NSI	this study
YQs4	*P*_Trc_: *araE*, *P*_BAD_-*mtgfp* integrated at NSI	this study
YQs5	*P*_J23119_: *araA-araB-araD* integrated at NSII	this study
YQs6	YQs5, *P*_BAD_-*mtgfp* integrated at NSI	this study

### Plasmid construction

The plasmids and primers used in this study were summarized in Tables S1 and S2.

All plasmids used in this study were constructed based on *S*. *elongatus* specialized plasmid pAM2991 (Addgene #40248)^36^ and pAM1573 (Addgene #40239)^34^.

The *araE*, *araA*, *araB* and *araD* genes were amplified from *E*. *coli* MG1655 genomic DNA. The *araE* gene amplified by primers Q1 and Q2 then digested with EcoRI, the *mtgfp* gene amplified by primers Q50 - Q51 then digested with EcoRI and BamHI, the *araE-mtgfp* fragment fused by primers Q3 - Q6 then digested with EcoRI, were respectively inserted into the multicloning sites (MCS) of pAM2991 to construct pYQ1, pYQ2 and pYQ3 plasmids. The *mtgfp* gene amplified by Q7 and Q8, digested with EcoRI and HindIII, then inserted into the MCS of pBAD backbone plasmid (Addgene #61284)^37^, generating pYQ4. The *araC-*P_BAD_ expression cassette containing *mtgfp* gene amplified by primers Q9 and Q10 from plasmid pYQ4 was inserted into the NotI site of pAM2991 and pYQ1 to create plasmid pYQ5 and pYQ6. The pYQ7 plasmid containing *araA*, *araB* and *araD* genes was created based on pcrRNA.con (Addgene #61285)^37^ by Q14 - Q21 primers using the Gibson Assembly method. Then the fragment from pYQ7 containing the constitutive promoter P_J23119_, *araBAD* genes and terminator was amplified by Q40 and Q41 primers, digested with XhoI and XbaI and inserted into the MCS of pAM1573 to build pYQ8 plasmid.

### Transformation of *S*. *elongatus*

Natural Transformation of *S*. *elongatus* was carried out as previously described^38^. Briefly, strains were transformed by incubating cells at mid-log phase (OD_730_ of 0.4 to 0.7) with 2 μg of plasmid DNA at 30 °C with gentle shaking overnight in the dark. Then, the culture was spread on BG-11 agarose plates with the required antibiotics to select transformants. Mutations were confirmed by colony PCR and DNA sequencing (BGI, Beijing, China) to verify the integration of targeting fragments at NSI and NSII sites of the chromosome. The constructed *S*. *elongatus* strains were list in Table 1.

### Confocal Microscopy

Confocal laser scanning microscopy images were taken on an Olympus FV1000 confocal laser scanning microscope (Olympus, Japan) with a 60× oil immersion objective and with 100-μm confocal pinhole aperture. Both the mtGFP fluorescence and chlorophyll fluorescence were excited at 488 nm. Emission was recorded simultaneously under the same exposure time: green fluorescence was collected using a 500–520 band pass filter and red chlorophyll auto-fluorescence was recorded using a 650 nm long-pass filter. Cells were placed in glass-bottom dishes for imaging.

### Fluorescence assay on plate reader

To test the mtGFP expression levels in *S*. *elongatus*, cells harvested at exponential phase were diluted to an OD_730_ of 0.1 to 0.2 in 100 mL BG-11 medium with various concentrations of inducers added. All fluorescence assays were conducted using a PerkinElmer EnSpire plate reader, the fluorescence excitation and emission wavelengths were set to 488 and 509 nm, respectively. All samples were tested by adding 200 μL broth in black 96-well plates. Specifically, to avoid the effect of IPTG on the P_BAD_ promoter, *S*. *elongatus* YQs4 cells pre-induced with IPTG were washed with BG-11 media for 3 times, then diluted to the same density.

To test mtGFP expression levels under non-induced condition in *E*. *coli*, strain BW25113, YQe1 and YQe2 were prepared by isolating and then culturing in LB medium overnight. After 1% inoculated into 50 mL fresh LB media supplemented with spectinomycin at 37 °C for 8 h, cells were then centrifuged at 6,000 g for 10 min and resuspended in 500 μL phosphate buffered saline (PBS) for measurement. All samples were tested by adding 200  μL suspension in black 96-well plates.

### *L*-arabinose consumption assay

Residual *L*-arabinose in culture supernatant was determined by orcinol method as previously described^39^. Samples were prepared by filtering the BG-11 broth with 0.22 μm cellulose acetate membrane. Color-developing regent was prepared by dissolving orcinol in concentrated HCl-AcOH (1:3, v/v) mixture to the concentration of 0.1%, and 99 mL of this solution was mixed with 1 mL FeCl_3_ (1 M) just before the use. Reaction mixture containing 1 mL sample and 4 mL color-developing regent was heated in boiling water bath for 30 min and then immediately cooled to room temperature for wavelength measurement. A UV-2000 spectrophotometer (UNICO, USA) was used to measure the wavelength, and the detective wavelength was set to 665 nm.

### Determination of Chlorophyll Content

Chlorophyll content was determined by methanol method as previously reported^40^. After 5 days cultivation in constant light condition, *S*. *elongatus* culture broth (1 mL) was centrifuged at 10,000 g for 10 min and cells were re-suspended in 100 μL distilled water. Methanol (100%, 900 μL) was added with thoroughly mixing, then the mixures were incubated at room temperature for 10 mins in the dark condition. After centrifugated at 10,000 g for 5 min, the supernatant was taken for chorophyll a measurement at the absorbance of 665 nm. The extinction coefficient for chlorophyll a is 13.4 mL/mg.

### Statistical Analysis

All statistical analysis was carried out in the SPSS software and the differences between data were evaluated using independent-samples *t*-test with P < 0.05 as significant. Error bars represent standard deviation in at least triplicate.

### Data Availability

There is no large-scale data associated with this manuscript. All constructs and strains are available on request.

## Electronic supplementary material

Supplementary Information

## References

[1] Oliver JW, Atsumi S (2014). Metabolic design for cyanobacterial chemical synthesis. Photosynth. Res..

[2] Hays SG, Ducat DC (2015). Engineering cyanobacteria as photosynthetic feedstock factories. Photosynth. Res..

[3] Niederholtmeyer H, Wolfstädter BT, Savage DF, Silver PA, Way JC (2010). Engineering cyanobacteria to synthesize and export hydrophilic products. Appl. Environ. Microbiol..

[4] Oliver JW, Machado IM, Yoneda H, Atsumi S (2013). Cyanobacterial conversion of carbon dioxide to 2, 3-butanediol. Proc. Natl. Acad. Sci. USA.

[5] Ruffing, A. M. & Kallas, T. Cyanobacteria: the Green *E. coli*. *Front. Bioeng. Biotechnol*. **4**, 7, 10.3389/fbioe.2016.00007 (2016). 10.3389/fbioe.2016.00007 PMC4735371 26870727

[6] Zhou J, Zhu T, Cai Z, Li Y (2016). From cyanochemicals to cyanofactories: a review and perspective. Microb. Cell Fac..

[7] Berla BM (2013). Synthetic biology of cyanobacteria: unique challenges and opportunities. Front. Microbiol..

[8] Ramey CJ, Barón-Sola AN, Aucoin HR, Boyle NR (2015). Genome engineering in cyanobacteria: where we are and where we need to go. ACS Synth. Biol..

[9] McEwen JT, Machado IM, Connor MR, Atsumi S (2012). Engineering *Synechococcus elongatus* PCC7942 to grow continuously in diurnal conditions. Appl. Environ. Microbiol..

[10] Kanno M, Carroll AL, Atsumi S (2017). Global metabolic rewiring for improved CO_2_ fixation and chemical production in cyanobacteria. Nat. Commun..

[11] Kanno M, Atsumi S (2016). Engineering an obligate photoautotrophic cyanobacterium to utilize glycerol for growth and chemical production. ACS Synth. Biol..

[12] Heidorn T (2011). Synthetic biology in cyanobacteria engineering and analyzing novel functions. Methods Enzymol..

[13] Agrawal GK, Kato H, Asayama M, Shirai M (2001). An AU-box motif upstream of the SD sequence of light-dependent psbA transcripts confers mRNA instability in darkness in cyanobacteria. Nucleic Acids Res..

[14] Michel KP, Pistorius EK, Golden SS (2001). Unusual Regulatory Elements for Iron Deficiency Induction of the idiA Gene of *Synechococcus elongatus* PCC 7942. J. Bacteriol..

[15] Erbe JL, Adams AC, Taylor KB, Hall LM (1996). Cyanobacteria carrying ansmt-lux transcriptional fusion as biosensors for the detection of heavy metal cations. J. Ind. Microbiol. Biotechnol..

[16] Geerts D, Bovy A, de Vrieze G, Borrias M, Weisbeek P (1995). Inducible expression of heterologous genes targeted to a chromosomal platform in the cyanobacterium *Synechococcus* sp. PCC 7942. Microbiology.

[17] Amirzada MI (2014). Cost-effective production of recombinant human interleukin 24 by lactose induction and a two-step denaturing and one-step refolding method. J. Ind. Microbiol. Biotechnol.

[18] Mutsuda M, Michel K-P, Zhang X, Montgomery BL, Golden SS (2003). Biochemical Properties of CikA, an Unusual Phytochrome-like Histidine Protein Kinase That Resets the Circadian Clock in *Synechococcus elongatus* PCC 7942. J. Biol. Chem..

[19] Atsumi S, Higashide W, Liao JC (2009). Direct photosynthetic recycling of carbon dioxide to isobutyraldehyde. Nat. Biotechnol..

[20] Ducat DC, Avelar-Rivas JA, Way JC, Silver PA (2012). Rerouting carbon flux to enhance photosynthetic productivity. Appl. Environ. Microbiol..

[21] McEwen JT, Kanno M, Atsumi S (2016). 2, 3 Butanediol production in an obligate photoautotrophic cyanobacterium in dark conditions via diverse sugar consumption. Metab. Eng..

[22] Horng YT (2010). Enhanced polyhydroxybutyrate (PHB) production via the coexpressed *phaCAB* and *vgb* genes controlled by arabinose P_BAD_ promoter in *Escherichia coli*. Lett. Appl. Microbiol..

[23] Lee SK (2007). Directed evolution of AraC for improved compatibility of arabinose-and lactose-inducible promoters. Appl. Environ. Microbiol..

[24] Guzman L-M, Belin D, Carson MJ, Beckwith J (1995). Tight regulation, modulation, and high-level expression by vectors containing the arabinose P_BAD_ promoter. J. Bacteriol..

[25] Khlebnikov A, Skaug T, Keasling JD (2002). Modulation of gene expression from the arabinose-inducible araBAD promoter. J. Ind. Microbiol. Biotechnol..

[26] Immethun CM (2017). Physical, chemical, and metabolic state sensors expand the synthetic biology toolbox for *Synechocystis* sp. PCC 6803. Biotechnol. Bioeng..

[27] Morgan-Kiss RM, Wadler C, Cronan JE (2002). Long-term and homogeneous regulation of the *Escherichia coli* araBAD promoter by use of a lactose transporter of relaxed specificity. Proc. Natl. Acad. Sci. USA.

[28] Liu J-J (2016). Metabolic engineering of probiotic *Saccharomyces boulardii*. Appl. Environ. Microbiol..

[29] Thomas C, Andersson CR, Canales SR, Golden SS (2004). PsfR, a factor that stimulates psbAI expression in the cyanobacterium *Synechococcus elongatus* PCC 7942. Microbiology.

[30] Hansen LH, Knudsen S, Sørensen SJ (1998). The effect of the *lacY* gene on the induction of IPTG inducible promoters, studied in *Escherichia coli* and *Pseudomonas fluorescens*. Curr. Microbiol..

[31] Latifi A, Ruiz M, Zhang C-C (2009). Oxidative stress in cyanobacteria. FEMS Microbiol. Rev..

[32] Jojima T, Omumasaba CA, Inui M, Yukawa H (2010). Sugar transporters in efficient utilization of mixed sugar substrates: current knowledge and outlook. Appl. Microbiol. Biotechnol..

[33] Englesberg E, Squires C, Meronk F (1969). The *L*-arabinose operon in *Escherichia coli* B/r: a genetic demonstration of two functional states of the product of a regulator gene. Proc. Natl. Acad. Sci. USA.

[34] Andersson CR (2000). Application of bioluminescence to the study of circadian rhythms in cyanobacteria. Methods Enzymol..

[35] Rippka R, Deruelles J, Waterbury JB, Herdman M, Stanier RY (1979). Generic assignments, strain histories and properties of pure cultures of cyanobacteria. Microbiology.

[36] Ivleva NB, Bramlett MR, Lindahl PA, Golden SS (2005). LdpA: a component of the circadian clock senses redox state of the cell. EMBO J..

[37] Luo ML, Mullis AS, Leenay RT, Beisel CL (2014). Repurposing endogenous type I CRISPR-Cas systems for programmable gene repression. Nucleic Acids Res..

[38] Golden SS, Brusslan J, Haselkorn R (1987). Genetic engineering of the cyanobacterial chromosome. Methods Enzymol..

[39] Tomoda M (1963). Colorimetric Determination of Pentoses. IV. Determination with Orcinol Reagent. Chem. Pharm. Bull..

[40] Porra R, Thompson W, Kriedemann P (1989). Determination of accurate extinction coefficients and simultaneous equations for assaying chlorophylls a and b extracted with four different solvents: verification of the concentration of chlorophyll standards by atomic absorption spectroscopy. Biochim. Biophysica. Acta Bioenerg..

