# Supplementary material for: AraBAD Based Toolkit for Gene Expression and Metabolic Robustness Improvement in Synechococcus elongatus
Source: Sci Rep. 2017 Dec 22;7:18059. doi: 10.1038/s41598-017-17035-4 (PMC5741739; doi:10.1038/s41598-017-17035-4)
Supplement: Supplementary file 1 — Supplementary Information [file 41598_2017_17035_MOESM1_ESM.pdf]

Supplementary Information

AraBAD Based Toolkit for Gene Expression and Metabolic Robustness Improvement in *Synechococcus elongatus*

Yi-Qi Cao[1], Qian Li[1], Peng-Fei Xia[1], Liu-Jing Wei[2], Ning Guo[1], Jian-Wei Li[1], Shu-Guang Wang[1,*]

[1]School of Environmental Science and Engineering, Shandong University, 27 Shanda Nanlu, Jinan 250100, China

[2]State Key Laboratory of Bioreactor Engineering, East China University of Science and Technology, 130 Meilong Road, Shanghai 200237, China

*Correspondence and materials should be addressed to S.-G.W. (email: wsg@sdu.edu.cn)

Figure legends

Figure S1. Fluorescence of eGFP and mtGFP led by P_{BAD} promoter in *S. elongatus* after inducing with 2 g/L *L*-arabinose for 2 days.

Figure S2. Standardized fluorescent intensity (RFU/OD_{600}) of *E. coli* BW25113, YQe1 (P_{BAD}) and YQe2 (P_{Trc}) under non-induced condition. An asterisk (*) represents a statistical difference ($p < 0.05$).

Figure S3. Fluorescence images of *S. elongatus* strains. (A) YQs1 and (B) YQs2 after inducing with 20 g/L *L*-arabinose and 5 mM IPTG for 2 days, respectively.

Figure S4. Growth curve of *S. elongatus* in continuous light condition with or without 2 g/L *L*-arabinose for 10 days.

Table legends

Table S1. Plasmids used in this study

Table S2. Primers used in this study

Figures

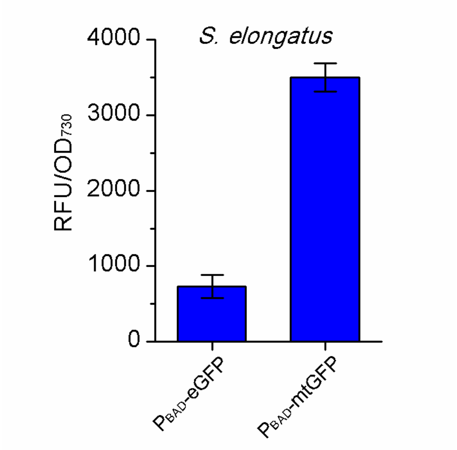

Figure S1. Fluorescence of eGFP and mtGFP led by P_{BAD} promoter in *S. elongatus* after inducing with 2 g/L *L*-arabinose for 2 days.

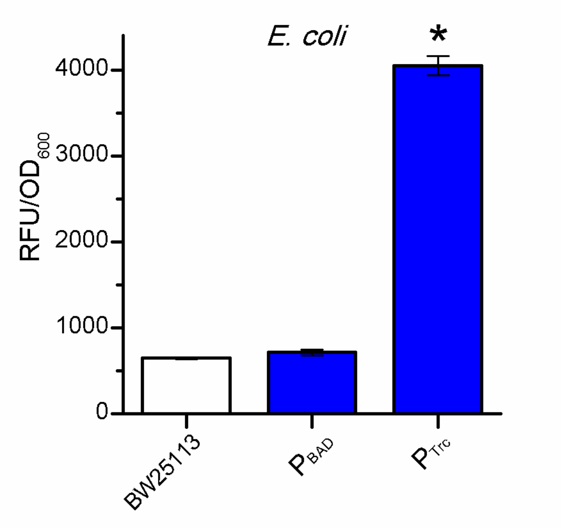

Figure S2. Standardized fluorescent intensity (RFU/OD_{600}) of *E. coli* BW25113, YQe1 (P_{BAD}) and YQe2 (P_{Trc}) under non-induced condition. An asterisk (*) represents a statistical difference ($p < 0.05$).

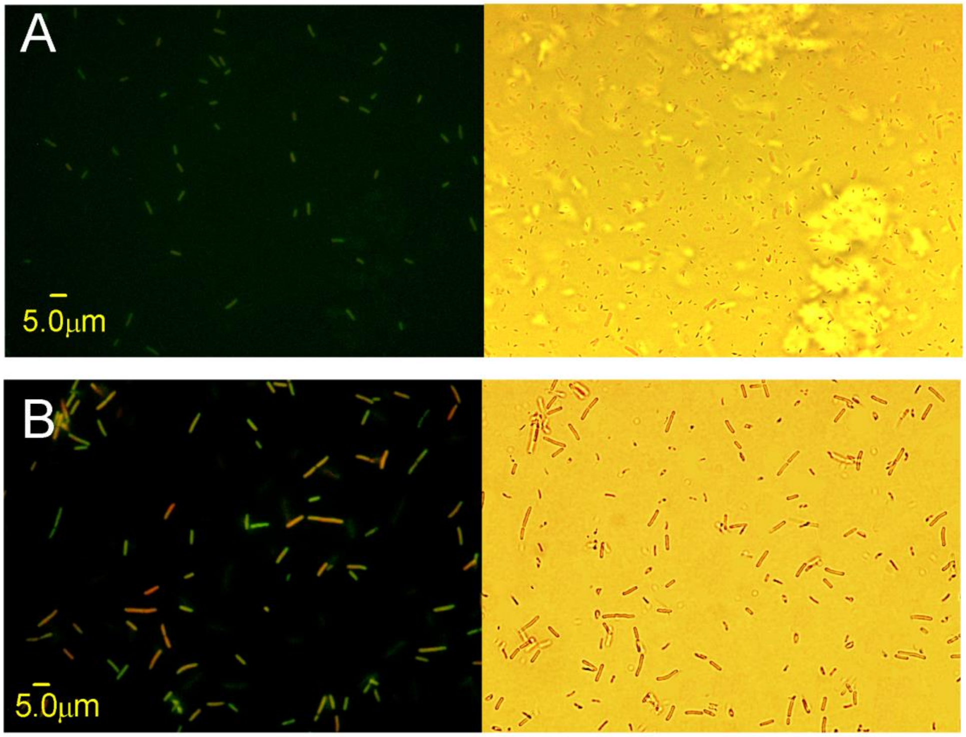

Figure S3. Fluorescence images of *S. elongatus* strains. (A) YQs1 and (B) YQs2 after inducing with 20 g/L *L*-arabinose and 5 mM IPTG for 2 days, respectively.

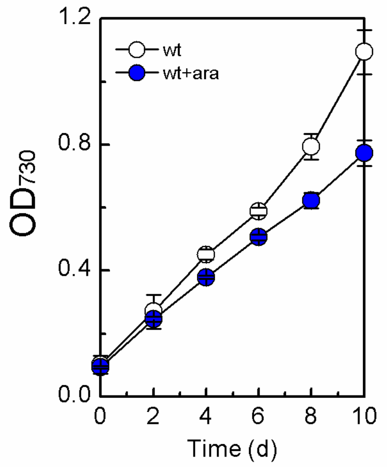

Figure S4. Growth curve of *S. elongatus* in continuous light condition with or without 2 g/L *L*-arabinose for 10 days.

Tables

Table S1. Plasmids used in this study.

**Plasmids**	**Genotypes**	**Sources**
pAM2991	NSI targeting vector; P_{Trc}; Sp^{R}	Addgene #40248
pAM1573	NSII targeting vector; Cm^{R}	Addgene #40239
pcrRNA.ind	*araC*-P_{BAD}; $\mathrm{Amp}^{\mathrm{R}}$	Addgene #61284
pcrRNA.con	P_{J23119}; $\mathrm{Amp}^{\mathrm{R}}$	Addgene #61285
pYQ1	pAM2991, but P_{Trc}: *araE*	This study
pYQ2	pAM2991, but P_{Trc}: *mtgfp*	This study
pYQ3	pAM2991, but P_{Trc}: *araE-mtgfp*	This study
pYQ4	pcrRNA.ind, but P_{BAD}: *mtgfp*	This study
pYQ5	pAM2991, but P_{BAD}: *mtgfp*	This study
pYQ6	pYQ1 containing P_{BAD}-*mtgfp*	This study
pYQ7	pcrRNA.con, but P_{J23119}: *araA*-*araB*-*araD*	This study
pYQ8	pAM1573, but P_{J23119}: *araA*-*araB*-*araD*	This study

Table S2. Primers used in this study**.**

Primers	Sequences (5’-3’)
Q1	ccggaattcatggttactatcaatacggaatctg
Q2	ccggaattctcagacgccgatatttctcaa
Q3	ccggaattcatggttactatcaatacggaatctgct
Q4	gtgaatagttcctcgccttttgacatgacgccgatatttctcaactt
Q5	aagttgagaaatatcggcgtcatgtcaaaaggcgaggaactatt
Q6	ccggaattcttacttgtataattcatccatgccca
Q7	ccggaattcatgtcaaaaggcgaggaactatt
Q8	cccaagcttttacttgtataattcatccatgccca
Q9	aaggaaaaaagcggccgcttatgacaacttgacggctacatca
Q10	aaggaaaaaagcggccgcaaaaggccatccgtcaggat
Q14	catggcgcgaaggcatattacgggcagtaaaacgcagaagcggtctgataaaacagaatt
Q15	ccacacttcataattatcaaaaatcgtcat gtaccgagctcgaattcgctagcgctagca
Q16	tgctagcgctagcgaattcgagctcggtacatgacgatttttgataattatgaagtgtggtttgtcattggcagc
Q17	gccaaaatcgaggccaattgcaatcgccatttagcgacgaaacccgtaatacacttcgttccagcgcag
Q40	ccgctcgagtgctcatgtttgacagcttatcatcgat
Q41	tgctctagaaaaaggccatccgtcaggat
Q50	ccggaattcatgtcaaaaggcgaggaactatt
Q51	cgcggatccttacttgtataattcatccatgccca
